# Belimumab in childhood systemic lupus erythematosus: A review of available data

**DOI:** 10.3389/fimmu.2022.940416

**Published:** 2022-07-27

**Authors:** Feng Chen, Ying Zheng, Xinying Chen, Zhanfa Wen, Youjia Xu, Jinghua Yang, Kaisi Xu

**Affiliations:** ^1^The Second Clinical Medical College, Guangzhou University of Chinese Medicine, Guangzhou, China; ^2^Department of Pediatrics, The Second Affiliated Hospital of Guangzhou University of Chinese Medicine, Guangzhou, China; ^3^Xiaorong Luo’s Renowned Expert Inheritance Studio, Guangdong Provincial Hospital of Chinese Medicine, Guangzhou, China

**Keywords:** Belimumab, childhood systemic erythematosus (cSLE), targeted therapy, pediatric lupus, immunology

## Abstract

**Introduction:**

Childhood systemic lupus erythematosus (cSLE) is a complex multisystem autoimmune disease. In 2019, belimumab was approved for the clinical treatment for cSLE, making it the only biological agent approved for cSLE children aged 5 and older in 60 years.

**Objective:**

To review emerging evidence on belimumab in cSLE published up to April 2022, so as to provide information for clinical decision-making.

**Method:**

A comprehensive search of relevant publications up to the date of April 2022 in PUBMED, EMBASE, WOS, COCHRANE, ClinicalTrials.gov, CBM, CNKI and WANFANG was performed using the following criteria: (a) English and Chinese language studies; (b) RCT studies, cohort studies, or case-control studies; (c) patients with age <18; (d) Observational studies or case series studies contain more than 5 patients. All relevant literature was independently screened and reviewed by at least two reviewers and the obtained literature data were extracted and reviewed by two authors.

**Results:**

Five publications met the inclusion/exclusion criteria for cSLE: one randomized controlled trial, one retrospective cohort study, and three case series. There was a high degree of heterogeneity among several studies, and the availability of baseline and outcome data provided was uneven.

**Conclusion:**

At present, there is a lack of high-quality clinical trials of belimumab in the treatment of cSLE. Based on the current research, it is believed that the use of belimumab can inhibit cSLE activity, reduce the dose of corticosteroids and immunosuppressants, and delay kidney damage. Also it shows clinical benefit in alleviating symptoms of monogenic cSLE refractory to standard therapy. More studies are urgently needed to validate the clinical efficacy of belimumab in cSLE and to evaluate its long-term safety in pediatric populations to promote evidence-based practice.

## Introduction

Systemic lupus erythematosus (SLE) is an autoimmune disease caused by immune dysfunction. As the disease progresses, it can manifest as multisystem involvement (1). Most patients are adult females. Fundamentally, childhood systemic lupus erythematosus (cSLE) is the same disease as adult SLE with similar etiology, pathogenesis and laboratory findings, but the frequency and severity of certain clinical manifestations are different. cSLE is generally considered to be more severe than adult SLE, and the disease damage begins to accumulate earlier, with cheek erythema and renal damage as the main manifestations. About 15-20% of SLE patients develop before the age of 18, with the peak age of onset being 12.6 years old, and it is less common under the age of 5. Its incidence rate is 0.36-2.5 per 100,000 children, and the prevalence rate is 1.89-34.1 per 100,000 children. However, compared with Caucasian children, the incidence rate of SLE in Asian children is similar to that of other common childhood autoimmune diseases, such as juvenile idiopathic arthritis (2–6).

Compared with adult SLE, cSLE shows a more aggressive course, and is more active with the progression of the disease. Meanwhile, due to age factors, the drug burden of children is relatively heavy, and cSLE patients have a higher use rate of immunosuppressants (3, 7, 8). Since the disease itself and the treatment of cSLE can affect the physical, psychosocial and emotional growth and development of children, the management of cSLE is different from that of adults. Thus, its early diagnosis and intervention are particularly critical for the prognosis of the disease. Belimumab is a promising new drug for the treatment of cSLE, however, clinical experience of its use in children is very limited so far. Data on doses are sparse, and studies in pediatric patients only have few valid data available. The aim of this study was to look through the emerging evidence on belimumab in cSLE up to April 2022, and to provide a literature review of available data for clinical decision-making.

## Method

### Search strategy

With the subject terms of “systemic lupus erythematosus” and “belimumab”, a comprehensive search of relevant publications up to the date of April 2022 was conducted in PUBMED, EMBASE, WOS, COCHRANE, ClinicalTrials.gov, CBM, CNKI and WANFANG. Studies were eligible for inclusion if they met the following criteria: (a) English and Chinese language studies; (b) RCT studies, cohort studies, or case-control studies; (c) patients with age <18; (d) Observational studies or case series studies contain more than 5 patients.

Publications that met the following criteria were excluded: (a) studies on other diseases (eg, nephrotic syndrome in children, adult SLE alone); (b) systematic reviews, guidelines, comments and clinical trials with unavailable data; (c) case reports with the number of cases < 5; (d) meeting abstracts only or incomplete text; (e) studies with a focus on other interventions than belimumab; (f) adult studies that did not meet age criteria; (g) Animal experiments; (h) actually duplicated documents with different titles.

### Screening criteria

Two reviewers (*FC* and *YZ*) independently screened the titles and abstracts of all publications according to the predefined inclusion/exclusion criteria. Full texts of the articles were read if it could not be excluded by reading the abstracts. All relevant publications were independently reviewed by two reviewers.

### Data extraction

Data were extracted from the included studies. The extraction sheet included: general study information, study population characteristics, belimumab administration method, results, efficacy assessments, and categories of evidence. Data were collected by two authors and independently reviewed by two other authors. Face-to-face discussions were held for publications with any uncertainties.

### Results

**Figure 1:**
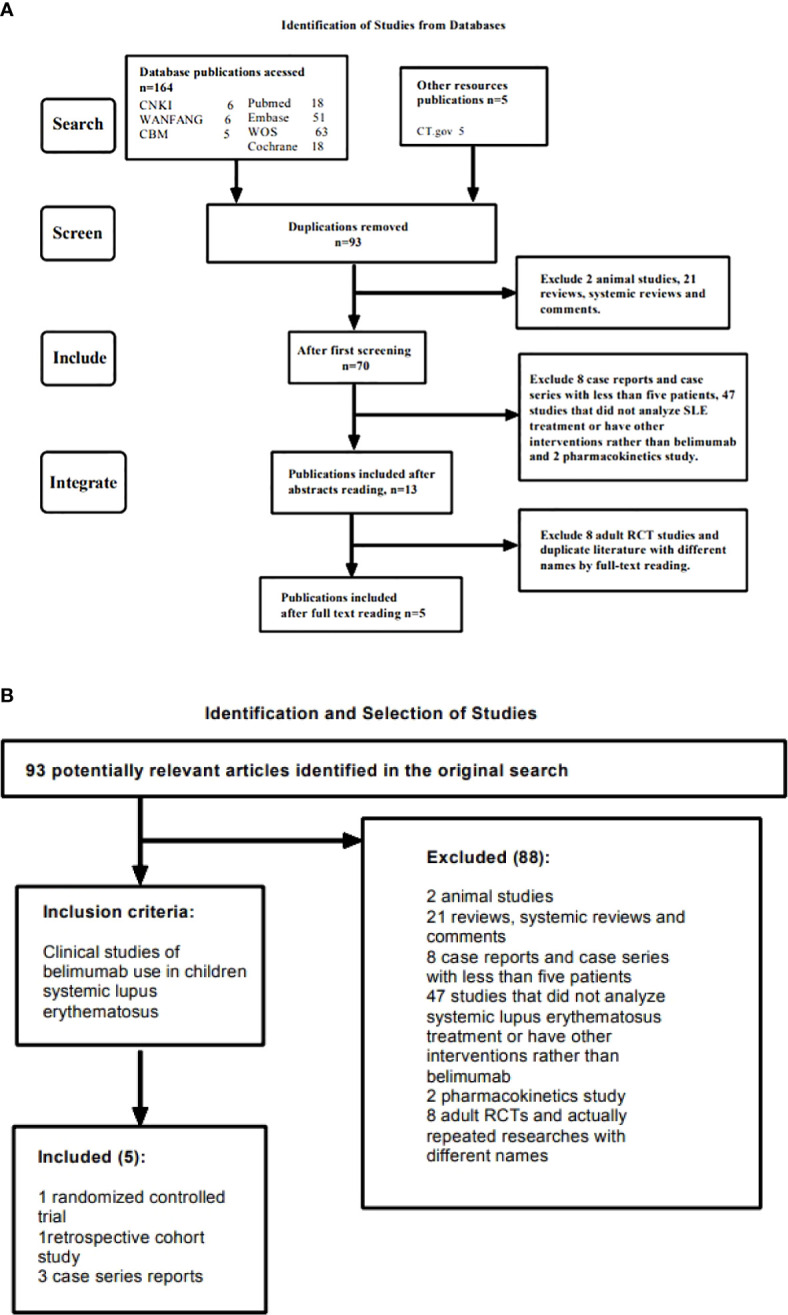
**(A)** Shows the searching and screening process of the study. **(B)** Shows the detailed inclusion and exclusion criteria in the study.

Both searches included the terms “systemic lupus erythematosus” and “belimumab”, therefore the same publications were captured by each search (n = 93), after exclusion of duplicates. After screening titles and abstracts, 79 studies that did not meet the inclusion criteria were excluded, the remaining 14 articles were read in full. After evaluating the publications according to the inclusion/exclusion criteria, 5 studies were finally determined to meet the complete criteria (**Figure 1**, **Table 1**).

**Table 1:** summarizes the five publications relating to the treatment of cSLE with BEL.

Study	Patients	Type of Study	Belimumab Regimen	Outcome Measures	Main result(s)	Safety Out comes	Level of evidence
*Hermine I Brunner et al.* USA.2020 (9)	Total 93 Placebo=40 BEL=53	Double-blind randomized placebo-controlled trial	52 weeks BEL 10mg/kg intravenous every 4 weeks	Primary outcomeSRI4 Major secondary efficacy outcomes: PRINTO/ACR, PGA, Parent-global, proteinuria	SRI4 responses were higher in BEL group than placebo group(52.8% vs. 43.6%); PRINTO/ACR in BEL group has higher proportion; Physician-GA, PGA and proteinuria were significantly improved in BEL group compared with placebo group.	AEs rates were similar between the two groups (79.2% vs. 82.5%).	1B
*Zeng Ping et al*. China.2021 (10)	Total 256 BEL-treated group=169 Traditional treatment =87	Nonrandomized retrospective cohort study	28 weeks 10mg/kg every 2 weeks for 3 doses, then every 4 weeks	SLEDAI-2K, LLDAS, GCs reduction, antibody	SLEDAI and antibody positive rate of both groups decreased, the difference being with no statistical significance; The proportion of reaching the status of LLDAS of the conventional group was lower than that of BEL group(8.8% vs. 40.5% P<0.001); The dosage of GCs in BEL group was significantly decreased.	AEs rate in BEL group was lower than that in conventional group.	2B
*Tao Jing et al.* China.2022 (11)	Total 8	Descriptive retrospective case series report	62 doses in total of 8 patients Unknown dose intravenous every 2 weeks for 3 doses, then every 4 weeks	Proteinuria, complement, antibody and other laboratory indicators	Most of children’s complement increased and antibody positive rate decreased, and only 2 children turned negative for proteinuria.	No AEs occurred during treatment.	4
*Lujayn Akbar et al*. KSA.2020 (12)	Total 6 (1 patient received BEL treatment as an adult)	Descriptive retrospective case series report	3-63months 10mg/kg every 2 weeks for 3 doses, then every 4 weeks	SLEDAI, PGA, antibody, complement, GCs reduction, urine protein/createnine ratio	Most of patients’ SLEDAI decreased, but PGA increased; Antibody, urine protein/creatinine ratio and GCs usage decreased significantly, but change of complement was not obvious.	1 patient died of sepsis, and no AEs occurred during treatment of the other patients.	1C
*Joyce S. Hui-Yuen et al*. USA.2015 (13)	Total 39 (with only 4 patients receiving BEL treatment before age 18)	Descriptive retrospective case series report	Unknown	GCs reduction, antibody, complement	Most cSLE patients reduced the GCs dosage, with complement increased and antibody positive rate decreased.	AEs rate was 16% (including adults).	4

BEL, Belimumab; PRINTO, Pediatric Rheumatology International Trials Organization; ACR, American College of Rheumatology; SRI4, SLE Response Index 4; SLEDAI, SLE Disease Activity Index; LLDAS, Lupus Low Disease Activity State; PGA, Parent-Global Assessment; GCs, Glucocorticoids; AEs, Adverse Events. Evidence level is assessed under OCEM-2001 standard.

## Overview of cSLE

### Pathogenesis of cSLE

The exact etiology of cSLE is not yet fully understood, but it is generally believed that the incidence of cSLE is related to genetic factors, neuroendocrine factors, immune abnormalities, environmental factors, infections, etc. Immune cells such as B cells, T cells, dendritic DC cells, inflammatory factors such as interleukin-6 (IL-6), mononuclear macrophages, tumor necrosis factor (TNF) and other factors are involved in the autoantigen immune response and inflammatory response of cSLE (14). In addition, the presence of pathogenic autoantibodies and overexpression of B cell activating factor (BAFF) are also considered to be associated with the disease. As an important factor in the survival and differentiation of B cells, BAFF can bind to receptors on B cells, thereby achieving the purpose of inhibiting B cell apoptosis and promoting proliferation and differentiation.

B cells, as antigen-presenting cells, which secretes pro-inflammatory cytokines and produce protective antigens, plays an important role in humoral immunity in healthy individuals; For SLE patients, B cell activation caused by various factors is the main pathogenesis, in which B cells are damaged and fail to recognize self-antigens. Thereby it produces antibodies against the self-antigens and triggers an overactive inflammatory response, resulting in extensive tissue damage (15, 16). In addition, monogenic cSLE has attracted much attention in recent years. Although it is rarer than common cSLE, its inheritance pattern and clinical manifestations are significantly different from those of common cSLE, which will be discussed later (17).

### Treatment of cSLE

Glucocorticoids are still the most commonly used basic drugs in the treatment of cSLE. Studies have shown that cSLE has higher frequency of immunosuppressive use and dosage of glucocorticoids than adults SLE (8). However, it is emphasized that an individualized medication plan should be formulated according to the specific situation of children. And the drug dose should be adjusted in time after the disease is relieved, so as to avoid the recurrence of the disease caused by excessive dose reduction, as well as the side effects of the drug caused by long-term use; If the disease involves important organs, immunosuppressants such as azathioprine (AZA), methotrexate (MTX), cyclophosphamide (CYC) or mycophenolate mofetil (MMF) are recommended at the initial stage of treatment. Being beneficial for the success rate of induction of remission, immunosuppressants improve organ involvement as well, but the side effects of them are still unavoidable. The Single Hub and Access point for pediatric Rheumatology in Europe (SHARE) recommends that all children with cSLE start regular use of hydroxychloroquine (HCQ) at an early stage of the disease, however, there is no new evidence from pediatric trials to support this recommendation yet (18–20).

In recent years, a variety of targeted drugs have been developed based on the pathogenesis of SLE, such as atacicept and telitacicept, targeted drugs for B cells. Besides, there are targeted drugs for the type I interferon pathway, anifrolumab and BIIB059, and baricitinib in the signal transducer and activator of transcription pathway of the Janus kinase, ustekinumab in regulating inflammatory factors, etc. (21). As an emerging treatment method, biological agents can avoid the long-term use of glucocorticoids alongside the corresponding side effects. Currently, the commonly used biologics for cSLE mainly include rituximab (RTX) and belimumab (BEL). Based on the positive results of the randomized controlled clinical trial “PLUTO” (9), BEL has become a promising new drug for the treatment of cSLE in recent years, and is the only biologic approved for patients aged 5 and older of SLE in the past 60 years.

## Overview of BEL

### Mechanism of BEL

BEL is a recombinant human monoclonal antibody that combines and neutralizes BAFF, which is developed as a new biological treatment for SLE. B cells are an important factor in connecting the two. According to studies, the level of BAFF in patients with SLE is much higher than that of healthy people, and BAFF level is correlated to SLE disease activities. BEL can bind and antagonize soluble BAFF to prevent it from combining with other receptors (22–24).

The two large-scale multi-centered RCTs among adults, BLISS-52 and BLISS-76, showed that BEL group’s SELENA-SLEDAI score, PGA, BILAG, glucocorticoids use and peripheral blood B cells, anti-dsDNA and complement were significantly improved. Besides, most outcomes have reached statistical significance compared with the comfort group. RTX is another candidate therapy targeted at B -cells, in addition to BEL. Although the ability of RTX to consume B -cells is far better than BEL, neither its primary nor secondary outcomes in clinical trials have reached statistical significance. Based on these, BEL was approved as the first new therapy for SLE in 2011 (9, 22, 25). At the same time, based on the adult SLE RCT mentioned above, a “PLUTO” study carried out by GlaxoSmithgKline for cSLE showed that the effectiveness and safety of BEL in cSLE patients were consistent with the treatment of adult SLE. Therefore, BEL was approved for the treatment of SLE in children in 2019 by FDA.

### Pharmacokinetics of BEL in cSLE

Pharmacokinetic models were established in 53 children treated with BEL in the PLUTO study, and samples were provided for PK assessment and exposure response analysis at each treatment. The parameters included in the analysis were maximum serum drug concentration (Cmax), minimum serum drug concentration (Cmin), stable distribution concentration (Cavg_ss), first phase elimination half-life (T1/2α), second (terminal) phase elimination half-life (T1/2β), steady-state volume of distribution (Vss), final phase volume of distribution (Vz), clearance (CL) and area under serum drug concentration-time curve (AUCss). After the recording and analysis of the above values, in the group of children treated with 10 mg/kg BEL every 4 weeks, the Cmin was 56.2ug/ml, the Cmax was 325ug/ml, the CL was 158ml/day, and the terminal half-life was 16.3 days with a distribution half-life of 0.8 days. In the comparison of serum drug concentrations, it was found that children were consistent with adults in the study (Cmin 55.6ug/ml, Cmax 313ug/ml); the steady-state PK curve simulated from children’s data was consistent with adult’s curve. The exposure distribution of all age groups in children shows a high degree of overlap in the charts and is consistent with that of adults (9, 26).

However, since the data on the pharmacokinetics of BEL were derived from Chinese adults, children from the same ethnic group were not included. Therefore, in order to prove the applicability of the medication regimen in cSLE population, Chinese scholars used the previous research data of BEL to compare the pharmacokinetics and exposure distribution between adult and pediatric patients of Chinese and non-Chinese ethnic groups. Thus, steady-state BEL exposure was predicted in cSLE children receiving 10 mg/kg every 4 weeks. In examining the effects of covariates such as age and race on BEL exposure, no significant differences were found in exposure between races. After the treatment of adult and pediatric patients, the first and third quartiles of Cavg_ss in the pediatric patient population were 78.2 and 105.8 mg/L. The adult distribution interval was calculated with 80-125%. The first quartile of the total adult population and Northeast Asian adults was 64.8-101.3 mg/L, 60.6-94.8 mg/L, and the third quartile was 89.0-139.0 mg/L and 78.4-122.5 mg/L (27). In terms of interval calculation of distribution, the classical 80-125% is selected as the elastic interval based on the provisions of bioequivalence limits. According to the numerical results, it is considered that the two distributions are similar (28–30). Combining the above Cavg_ss values ​​for children and adults, it shows the similarity in exposure distribution between the two. Therefore, the 10 mg/kg weight-proportional dosing regimen is also applicable to Chinese patients with cSLE.

## Progress of BEL in the treatment of cSLE

### Effect on disease symptom score

Due to the complexity of clinical symptoms and serological manifestations of cSLE, it is a highly heterogeneous disease. The severity of the disease also varies over time, with periods of inactive alternating with periods of relapse. In addition to such heterogeneity, complications and infections further complicate the clinical presentation of the disease. Therefore, to facilitate the assessment of disease activity in clinic, different disease scoring tools have emerged (31–33). The most widely used include SLE Disease Activity Index (SLEDAI), Physicians Global Assessment (PGA), British Isles Lupus Assessment Group (BILAG), as well as Systemic Lupus Activity Measurement (SLAM) (34).

The Pediatric Lupus Trial of Belimumab Plus Background Standard Therapy (PLUTO) is the first double-blind, placebo-controlled randomized trial in children with active SLE. In this study, a total of 93 children (aged 5-17 years old) were assigned to the intervention group and the control group with 53 and 40 children respectively. The intervention group was administered at a dose/frequency of 10mg/kg every 4 weeks for 52 weeks. Compared with the control group, the intervention group had an average advantage of 5.3% higher in the improvement of SLEDAI (9). In a multi-center retrospective study, *Zeng et al.* conducted a cohort analysis of 256 children with cSLE from 37 centers and found that the SLEDAI score of children in BEL group and the traditional drug group both decreased gradually with extension of time, however the mean SLEDAI scores at week 28 were similar between the two treatment regimens and the difference was not statistically significant (10).

According to *Zeng’s* report, at the 28th week of monitoring, the proportion of children who achieved lupus low disease activity state (LLDAS) in BEL treatment group (40.5%) was higher than that in traditional drug treatment group (8.8%) (10). In terms of PGA score, in PLUTO study, the average PGA score of BEL group was 7.7% lower than that of the control group (9); As for BILAG score, children treated with BEL in PLUTO study had a significant improvement in BILAG musculoskeletal score compared with placebo group, while no significance difference was found in BILAG skin and mucosa score. In the improvement of SRI scores, BEL enabled a higher proportion of children in the intervention group (52.8%) to achieve the primary efficacy endpoint of SRI-4 compared to the control group (43.6%) (9). In addition,.

Overall, BEL appears to have a positive effect on improving disease scores in children with cSLE, but in higher-quality evidence-based study, the combination of BEL does not show an impressive advantage compared with conventional medicine. Still, it showed potential for faster control of lupus activity to a lower state.

### Effect on laboratory results

Based on the B-cell targeting mechanism of BEL and the immune overregulation and organ damage of SLE, we mainly focus on peripheral blood B cells, SLE-related antibodies such as ANA, anti-dsDNA, and complement. In terms of organ damage, kidney involvement is common, and we also pay attention to the situation of proteinuria.

It is mentioned above that B cells are the main target of BEL in the treatment of SLE. At week 52 of the PLUTO study, compared with the placebo group, the total number of peripheral blood B cells and immature B cells in the treatment group were significantly reduced. However, the study was based on measures developed for the treatment of adults, there was a lack of sufficient samples amount to support the statistical testing (9).

SLE is an autoimmune disease that forms immune complexes and antibodies, and it involves multiple systems. Therefore, serum antibodies are of great significance to its diagnosis and judgment of disease progression. ANA has high sensitivity and is the first choice for SLE screening, but its specificity is poor, and the sensitivity of it is easily affected by the quality of detection, which cause a risk of misdiagnosis and missed diagnosis. Since anti-dsDNA has higher specificity, combined detection of antibodies is considered to be more beneficial for the diagnosis of SLE (35, 36).

The PLUTO study showed that anti-dsDNA in the treatment group was lower than that in the placebo group, but the article did not provide detailed relevant data, thus the study did not support statistical testing (9). At present, most studies on the treatment of cSLE with BEL are based on case reports and retrospective studies. One of them is the multi-center real-world study reported by *Zeng* mentioned above. After 28 weeks of treatment, the study mainly observed the changes of ANA and anti-dsDNA before and after treatment in the serological aspect. At week 0 of enrollment, the positive rates of antibodies in the two groups were similar. After 28 weeks of treatment, the positive rates in both groups decreased. Compared with before, the difference was statistically significant, but there was no statistical difference between the two groups.

According to the data and charts of the two groups, except for a small number of children with no change or increase in SLE antibody after treatment, about 2/3 of patients showed a decrease or negative ANA titer after treatment. Both groups have nearly a half of the children turned negative for anti-dsDNA, and about a quarter of the children had anti-dsDNA titers lower than before. It can be seen that SLE antibodies were improved in varying degrees after BEL and traditional drug treatment in this group, but the difference between two groups did not reach statistical significance (10). In an observation of the efficacy on BEL treating cSLE reported by *Tao*, 8 children with active cSLE were selected as the observation objects. The SLE antibody in 4 children turned negative or decreased in positive rate after treatment, but limitations such as small sample size, short study duration, and lack of bias control (11).

The complement system is an important effect amplification system that is widely involved in the immune regulation of the body and mediates the injury response of immunopathology. Decreased complement levels are one of the common manifestations of SLE. According to the PLUTO study and the report of *Tao*, it can be seen that after BEL treatment, the complement level of most children was improved to varying degrees (9, 11).

Multisystem damage is one of the main manifestations of SLE. About 50%-75% of children with cSLE have kidney damage, which is 10%-30% higher than adult patients (2, 37). Therefore proteinuria is also one of the key points of attention. During the PLUTO study, the children with higher urinary protein levels in the two groups did not turn negative. While the children in the treatment group did not develop renal flares, indicating that the treatment of BEL played a certain role in delaying kidney damage (9). In another observational study, children with kidney damage before treatment turned negative and the positive rate of proteinuria could be reduced after treatment in some children, as well as some children without kidney damage developed urine protein at the end of treatment (11).

As far as the current studies are concerned, BEL shows reliable efficacy in inhibiting B cell activity, increasing complement levels in the body, and delaying kidney damage. But it requires more solid evidence to prove its effective in improving the positive rate of autoimmune antibodies and urinary protein.

### Effect on glucocorticoids use

Glucocorticoids, as the basic drugs for cSLE, have an indispensable position in current pediatric clinical practice. However, its significant side effects among childhood patients become a major obstacle to its long-term use. It is reported that children with cSLE are prone to accumulate more organ damage due to more intensive glucocorticoid application (6). Therefore, the ability of biological agents to reduce glucocorticoid dosage can indirectly promote the compliance of cSLE treatment.

*Zeng* reported that at the end point of observation at week 28, the oral glucocorticoids doses of children in both groups were reduced, yet the maintenance doses of the BEL treatment group were lower than that of conventional treatment group (10.69 ± 8.57 vs. 16.45 ± 8.3 mg/d, *p*<0.001) (10), which suggests BEL did help to achieve the goal of LLDAS with lower doses of corticosteroids. In the case series contained 8 children with cSLE reported by *Tao*, 6 children successfully reduced their glucocorticoids after receiving BEL (11). In a study of 39 patients with cSLE published in 2014 by *Hui-yuen*, the mean daily dose of prednisone decreased from 17 mg to 11 mg, and 35% of cSLE children discontinued glucocorticoids 6 months after initiation of BEL (13). It is worth mentioning that, in order to control variables, the PLUTO study was strictly controlled for any changes in treatment regimens, so no reduction in the dose of glucocorticoids was designed. Therefore, by the end of the study, the median glucose levels of children in the two experimental groups were strictly controlled. There was no decrease in corticosteroid use (9), this does not mean that the use of BEL has no effect on glucocorticoid dose in clinical practice though.

### Effect on immunosuppressants use

Immunosuppressive drugs including MMF, AZA, MTX, and CYC, are recommended for cSLE that cannot be reduced by oral prednisone or with persistent proteinuria, so as to control disease activity and help reduce glucocorticoids. However, its potential adverse reactions such as bone marrow toxicity, reproductive toxicity and potential carcinogenic risk cannot be ignored. In *Zeng’s* report, the author pointed out that the dosage of immunosuppressive drugs in combination did not change much in the BEL group, while the doses of HCQ and MMF in traditional drug treatment group increased significantly at the endpoint of observation, and the dose of CYC was significantly higher than that of the BEL group (10, 20, 38).

In addition, other studies did not mention the impact of BEL on the use of immunosuppressive drugs. Although the regular dosing of BEL may reduce the dosage of immunosuppressants according to current research in the real world, due to the limitation of the follow-up time of the study, further clinical studies are still needed to verify its related effects.

### Efficacy in children with monogenic cSLE

Monogenic SLE, first proposed by French scholar Alexandre Belot in 2012, is a type of disease with lupus-like phenotypes such as rash, nephritis and arthritis caused by homozygous or heterozygous mutations in a single gene. It has broken through the pathogenesis of SLE from a genetic point of view, and more than 30 pathogenic genes have been found so far. According to different mechanisms, pathogenic genes can be divided into 4 types: genetic deficiency of the complement system, excessive activation of type I interferon, destruction of the body’s tolerance and other pathways such as nucleic acid degradation, sensing, etc. (39–43). Monogenic SLE is a rare mutation, characterized by early onset, male prevalence, refractory and family history of autoimmune diseases. Moreover, its clinical manifestations are more diverse and the course of disease is unpredictable, representing a unique entity that may be different from common SLE (44).

As for treatment, there are limited available data on the treatment of cSLE with BEL, and the study of its application in monogenic SLE is even lacking. At present, a descriptive and retrospective series of case reports for monogenic cSLE reported by *Akbar* is the first study on the efficacy of BEL in monogenic cSLE. The study included 6 children. Except that one child was treated with BEL as an adult, the other five children were genetically confirmed as single gene defects and met the diagnostic criteria of SLE. Among them, 4 children had C1q deficiency and 1 child had deoxyribonuclease II (DNaseII) deficiency. BEL was added after the failure of intensive treatment with standard therapy. The dosage of BEL was 10 mg/kg every 2 weeks for 3 doses, then every 4 weeks. After BEL treatment, the skin and mucosal manifestations of all patients were significantly improved. As for arthritis, 2 patients completely resolved while another one with DNaseII deficiency has significant improvement from joint pain despite deformed joints. In terms of corticosteroid dosage, except for 1 patient had disease recurrence and corticosteroid supplementation due to irregular use of BEL, 1 patient achieved corticosteroid withdrawal, and during the follow-up, the average daily glucocorticoid dosage of 3 patients decreased significantly. After using BEL, 4 patients showed sustained improvement in SLEDAI score within a short period of time, but their PGA scores increased to varying degrees. As for parameters such as related antibodies and complement levels, except for the missing part of follow-up data, only a small number of patients achieved improvement, and most of them have no obvious changes or even worsened. For cSLE patients with nephritis, the combination of BEL and MMF had a positive effect on the urine protein-to-creatinine ratio, and this combination was also shown to be beneficial for inducing remission in lupus nephritis in adult studies. According to the above research results, it is suggested that the effectiveness of BEL in the treatment of monogenic cSLE is basically the same as that of ordinary cSLE. As for safety, except for 1 patient who lost follow-up after treatment and died of septic shock, the remaining patients had good tolerance to BEL, and no obvious adverse events occurred (12).

In conclusion, BEL had a positive effect on symptoms, glucocorticoids, and SLEDAI scores. BEL can be selected as an adjunct to the treatment of monogenic cSLE when traditional therapy does not work. However, the data on the treatment of monogenic cSLE are of great limitation, and factors such as small sample size, short follow-up time, and partial follow-up loss can’t be ignored. Thus, larger-scale prospective studies are needed to further verify the efficacy of BEL on monogenic cSLE.

## Safety of BEL in cSLE

PLUTO study, as the highest level of evidence-based medical study in the field to date, it provides detailed coverage of adverse reactions to BEL. Compared with 35% in the placebo group, the incidence of adverse events in the BEL group was 16.98%, including 1 case of pericardial effusion, 1 case of gastrointestinal vasculitis, 1 case of idiopathic intracranial hypertension and 2 cases of lupus nephritis (LN). In the placebo group, the main adverse events were infections. Yet the serious AEs haven’t been proved to be associated with the use of BEL. It is worth noting that the 3 cases of serious psychiatric adverse events (major depression, suicidal ideation and suicidal behavior) reported in the study all occurred in the placebo group. For other adverse events, there was no significant difference between the BEL group (67.92%) and the placebo group (67.5%) (9).

In the real-world study reported by *Zeng et al.*, unsatisfactory efficacy and different types of infections were the main reasons for discontinuation in the BEL group, including 3 cases of pulmonary fungus, 1 case of Pneumocystis carinii, and 2 cases of CMV infection. Among 169 patients with cSLE treated with BEL, there were no adverse events such as malignant tumor, suicide and death (10). Thus, the incidence of serious adverse events was lower in BEL group than in conventional drug group. However, the follow-up time in this study was only 28 weeks, which is insufficient for adverse reactions.

Previous clinical trials of BEL in adults, including BLISS-52 and BLISS-76, reported more frequent psychiatric adverse reactions, such as insomnia, anxiety and depression, in BEL treatment group than in traditional treatment group. In addition, 1 case of suicide was reported in each of BEL treatment groups at different doses (45). These reports illustrate the potential psychiatric risk associated with the use of BEL.The results of adverse events in current clinical trials of cSLE are not consistent with that of adults. Concerning high quality clinical trial of cSLE remains to be further developed, it is recommended that the mental state of children, especially those with active neuropsychiatric lupus, should be carefully evaluated before the use of BEL.

About the administration of BEL, all included studies among children were conducted intravenously except for the study of *Hui-Yuen* haven’t mentioned. Subcutaneous BEL among adults was proved safe and effective in improving SRI-4 response and decreasing symptoms (46). In a comparison review between weekly subcutaneous BEL and monthly intravenous BEL, the former appears to show similar safety yet better preference among adults group (47). Consider the compliance needed in a long-term treatment, further studies on subcutaneous BEL among children is required in the future.

## Conclusion

BEL is the first drug approved for cSLE in nearly half a century. Among the existing clinical studies of BEL, there are 1 RCT study with a 52-week observation period and 1 real-world study with a 28-month observation period while the rest were case series or case reports. Despite varying levels of evidence and observation periods, these literatures suggest that the use of BEL plays an active role in suppressing lupus activity, reducing steroids and immunosuppressants doses, delaying kidney damage, and relieving symptoms of monogenic cSLE that are refractory to standard therapy.

Due to the complexity of cSLE, the progression of organ damage is faster than that of adults, making the timeliness of clinical diagnosis and treatment rather essential. BEL has shown considerable clinical potential in the treatment of cSLE in recent years. However, due to the earlier use of biological agents than adults SLE, the course of BEL treatment is considered to be longer in children. In addition to a higher accumulated cost, it raised concern to the discontinuation because of unsatisfactory efficacy or different types of infection in a long-term treatment.

Despite the current positive results, more high-quality prospective randomized controlled trials with longer follow-up periods, as well as real-world data with larger sample sizes are urgently needed to verify its efficacy and safety in the treatment of cSLE.

## Author contributions

FC, YZ and XC made a substantial contribution to the conceptualization, methodology, analysis, and interpretation of the literature for this manuscript. FC and YZ drafted the first manuscript and FC reviewed the literature, corrected the manuscript and edited English style writing. ZW helped with the table and figures. YX and XC reviewed and critically revised the article. All authors discussed the results and commented on the manuscript. All authors contributed to the article and approved the submitted version.

## Funding

This study was supported by Xiaorong Luo's Renowned Expert Inheritance Studio of National Administration of Traditional Chinese Medicine (No.14GG2X02).

## Conflict of interest

The authors declare that the research was conducted in the absence of any commercial or financial relationships that could be construed as a potential conflict of interest.

## Publisher’s note

All claims expressed in this article are solely those of the authors and do not necessarily represent those of their affiliated organizations, or those of the publisher, the editors and the reviewers. Any product that may be evaluated in this article, or claim that may be made by its manufacturer, is not guaranteed or endorsed by the publisher.
